# Targeting the NLRP3 Inflammasome as a New Therapeutic Option for Overcoming Cancer

**DOI:** 10.3390/cancers13102297

**Published:** 2021-05-11

**Authors:** Sonia Missiroli, Mariasole Perrone, Caterina Boncompagni, Chiara Borghi, Alberto Campagnaro, Francesco Marchetti, Gabriele Anania, Pantaleo Greco, Francesco Fiorica, Paolo Pinton, Carlotta Giorgi

**Affiliations:** 1 Department of Medical Sciences, Section of Experimental Medicine and Laboratory for Technologies of Advanced Therapies (LTTA), University of Ferrara, 44121 Ferrara, Italy; sonia.missiroli@unife.it (S.M.); mariasole.perrone@unife.it (M.P.); caterina.boncompagni@unife.it (C.B.); paolo.pinton@unife.it (P.P.); 2 Department of Medical Sciences, Section of Obstetrics and Gynecology, University of Ferrara, 44121 Ferrara, Italy; borghi.chr@gmail.com (C.B.); pantaleo.greco@unife.it (P.G.); 3 Department of Medical Sciences, Section of General and Thoracic Surgery, University of Ferrara, 44121 Ferrara, Italy; alberto.campagnaro@edu.unife.it (A.C.); frances.marchetti@edu.unife.it (F.M.); gabriele.anania@unife.it (G.A.); 4 Department of Radiation Oncology and Nuclear Medicine, AULSS 9 Scaligera, Ospedale Mater Salutis di Legnago, 37045 Verona, Italy; francesco.fiorica@aulss9.veneto.it

**Keywords:** inflammation, NLRP3 inflammasome, inhibitors, target-therapy

## Abstract

**Simple Summary:**

NLRP3 inflammasome is a cytoplasmic multiprotein complex that assembles in response to cellular distress and promotes maturation and release of the inflammatory cytokines interleukin-1β and IL-18, which contribute to immune responses and inflammation. Aberrant NLRP3 activation has divergent roles in the pathogenesis of inflammation-associated diseases such as cancer, as it can have both protumorigenic and antitumorigenic effects in a context-dependent and tissue-specific manner. Therefore, the fine-tuning of the NLRP3 inflammasome in cancer cells, through a wide range of agents including, such as inhibitors, antagonists and monoclonal antibodies, has been suggested as a viable approach to cancer therapy.

**Abstract:**

Inflammasomes are multiprotein complexes that regulate the maturation and secretion of the proinflammatory cytokines interleukin-1beta (IL-1β and interleukin-18 (IL-18) in response to various intracellular stimuli. As a member of the inflammasomes family, NLRP3 is the most studied and best characterized inflammasome and has been shown to be involved in several pathologies. Recent findings have made it increasingly apparent that the NLRP3 inflammasome may also play a central role in tumorigenesis, and it has attracted attention as a potential anticancer therapy target. In this review, we discuss the role of NLRP3 in the development and progression of cancer, offering a detailed summary of NLRP3 inflammasome activation (and inhibition) in the pathogenesis of various forms of cancer. Moreover, we focus on the therapeutic potential of targeting NLRP3 for cancer therapy, emphasizing how understanding NLRP3 inflammasome-dependent cancer mechanisms might guide the development of new drugs that target the inflammatory response of tumor-associated cells.

## 1. Introduction

An association between inflammation and cancer development has long been appreciated, to the extent that the inflammatory response has been recognized to play a decisive role in the development and progression of cancer [1]. In the last decade, the term inflammasome was coined to describe a cytoplasmic multiprotein complex that recognizes signals of host cellular distress and activates proinflammatory caspases, which drive subsequent immune responses and inflammation [2]. Among the inflammasomes, NLRP3 is the most well studied and has been associated with the pathogenesis of multiple diseases, including neurodegenerative diseases (Alzheimer’s and Parkinson’s), atherosclerosis, hereditary cryopyrin-associated periodic syndromes (CAPSs), metabolic diseases and cancer [3]. Thus, considering the substantial role of NLRP3 in different inflammatory pathologies, it is not surprising that strategies targeting this inflammasome are hot topics of research. In this review, we will discuss the NLRP3 inflammasome as a double-edged sword in cancer, as it can have both protumorigenic and antitumorigenic effects not only in different cancers but also in a tissue-specific manner. Moreover, we will focus on current strategies for targeting the NLRP3 inflammasome, including a diverse range of agents, such as inhibitors, antagonists and monoclonal antibodies, which have potential for cancer treatment.

## 2. NLRP3 Inflammasome: Biology and Activation

The NLRP3 inflammasome is a critical component of the innate immune system and consists of NLRP3 (NLR family protein containing a pyrin domain 3), the adaptor apoptosis-associated speck-like protein (ASC, which contains a caspase activation and recruitment domain, CARD) and the effector pro-caspase 1. NLRP3 comprises three domains: An N-terminal pyrin domain (PYD), which interacts with the pyrin domain of ASC to initiate inflammasome assembly [4]; a central nucleotide-binding and oligomerization (NACHT) domain that is required for subsequent activation [5]; and a C-terminal leucine-rich repeat (LRR) domain, which has been recently demonstrated to be nonessential for NLRP3 inflammasome assembly [6]. ASC recruits and binds pro-caspase 1 via their shared domains, inducing autoproteolytic caspase-1 activation. Caspase-1 further induces the release of the inflammatory cytokines interleukin-1beta (IL-β) and interleukin-18 (IL-18) and cleaves gasdermin D (GSDMD), which in turn induces a form of programmed cell death known as pyroptosis [7,8].

**Figure 1:**
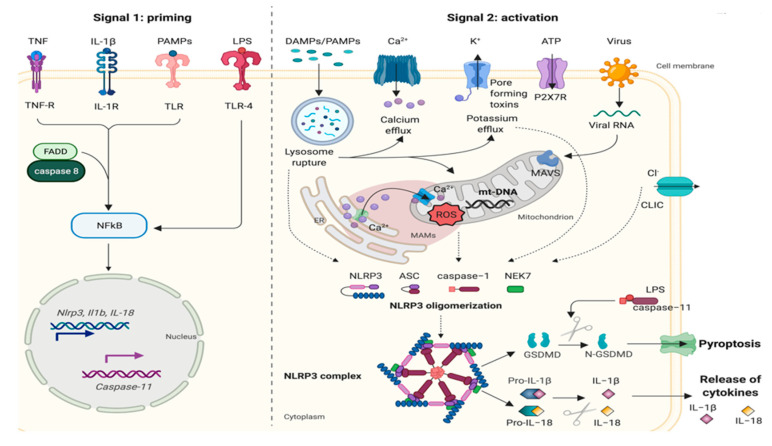
NLRP3 inflammasome priming and activation. The activation process of the NLRP3 inflammasome requires two main signals: (i) signal 1 (priming), which leads to the activation of the transcription factor NF-kappaB and the subsequent transcription of canonical and noncanonical NLRP3 inflammasome components; and (ii) signal 2 (activation), which is responsible for NLRP3 complex assembly and the subsequent release of inflammatory cytokines (IL-1β and IL-18). Priming is provided by exposure to pathogen-associated molecular patterns (PAMPs) such as lipopolysaccharide (LPS) or by endogenous cytokines that activate receptors at the cell membrane. The induction of NLRP3 expression during priming is controlled by FAS-associated death domain protein (FADD) and caspase-8. NLRP3 activation is provided by a plethora of stimuli, such as PAMPs or damage-associated molecular patterns (DAMPs), ATP, and viral RNA, that in turn trigger downstream signaling events such as mitochondrial damage, mitochondrial ROS production, lysosomal disruption, and ion (K^+^ and Ca^2+^) efflux. Mitochondrial antiviral signaling protein (MAVS) mediates the NLRP3 activation induced by RNA viruses. Excessive Ca^2+^ released from the ER causes mitochondrial dysfunction and is implicated in NLRP3 inflammasome activation. Chloride intracellular channel protein (CLIC)-mediated Cl^-^ efflux promotes the NEK7-NLRP3 interaction and subsequent NLRP3 inflammasome assembly. LPS can directly activate TLR4 to induce the transcription and activation of caspase-11, which in turn cleaves the pore-forming protein gasdermin D (GSDMD), which can induce pyroptosis. IL-18: interleukin-18; IL-1β: interleukin-1beta; IL-1R: interleukin 1 receptor; mt-DNA: mitochondrial DNA; ROS: reactive oxygen species; TLR: Toll-like receptor. Created with BioRender.com accessed on 30 March 2021.

Although a consensus for the precise localization of the NLRP3 inflammasome and its components has not yet been reached, several studies have indicated that, in resting conditions, NLRP3 resides in the endoplasmic reticulum (ER) and cytosol [9]. Upon activation, NLRP3 redistributes to mitochondria-associated membranes (MAMs) [10,11] and mitochondria [12]; however, one study proposed a strictly cytosolic localization of NLRP3 [13]. Although the NLRP3 inflammasome is the most studied and investigated, its activation mechanism remains unclear. To date, two steps have been proposed to underlie NLRP3 activation: priming and activation (Figure 1). The first step is triggered by the engagement of Toll-like receptors (TLRs) by their ligands or endogenous molecules, such as tumor necrosis factor (TNF) or IL-β, which leads to the transcription of NLRP3 and pro-IL-1β via the regulation of nuclear factor-kB (NF-κB) [14]. Moreover, recent studies have shown that the induction of NLRP3 expression during priming is also controlled by FAS-associated death domain protein (FADD) and caspase-8 [15].

In addition to control of NLRP3 activation via transcription, the priming step is also able to control inflammasome activation at the posttranscriptional level, as suggested by emerging evidence [16]. In particular, Schroder and colleagues demonstrated for the first time that priming of the NLRP3 inflammasome with short lipopolysaccharide (LPS) pretreatment could occur independently of NLRP3 protein expression induction [16].

It is well known that there are several NLRP3-activating stimuli and that they are heterogeneous; however, it is unclear why seemingly unrelated molecules can activate the same inflammasome. Given its heterogenous interaction partners, it is unlikely that NLRP3 physically interacts with its activators. All inflammasome activators induce cellular signaling that is sensed by the NLRP3 receptor. Numerous hypotheses for the origin of this signal have been proposed, including calcium (Ca^2+^) signaling, potassium (K^+^) efflux, effects of reactive oxygen species (ROS), lysosomal leakage, exposure to damage-associated molecular patterns (DAMPs) and pathogen-associated molecular patterns (PAMPs), and mitochondrial dysfunction [17,18].

Given that many stimuli, like nigericin, adenosine triphosphate (ATP) and particulates, lead to Ca^2+^ mobilization, different studies have suggested the involvement of Ca^2+^ signaling in NLRP3 inflammasome activation [19,20,21]. Ca^2+^ chelation using BAPTA-AM inhibits IL-1β release, but Ca^2+^ inhibition does not affect the activation of other inflammasomes, such as NLRC4 or AIM2 [19,22].

Several stresses trigger a change in the intracellular ionic composition that induces a conformational change in one of the inflammasome components, suggesting a role for K^+^ efflux in NLRP3 inflammasome activation [23,24]. Numerous studies agree that K^+^ efflux is a crucial event for inflammasome activation. Among these studies, Petrilli et al. demonstrated that in murine macrophages, NLRP3 inflammasome assembly occurs spontaneously at low K^+^ concentrations (below 90 mM) but is prevented at higher concentrations [24]. Many stimuli, including ATP and nigericin, act through a mechanism dependent on K^+^ efflux [25].

The involvement of mitochondrial dysfunction and ROS has been discussed for a long time, and ROS have been identified as important activators of the NLRP3 inflammasome [11,26]; in addition, several chemical inhibitors of ROS can prevent NLRP3 inflammasome activation [27,28]. Moreover, numerous stimuli inducing cell death and mitochondrial dysfunction promote mitochondrial DNA oxidation, which in turn activates inflammasomes [29].

In recent years, other cellular mechanisms have been proposed for inflammasome activation, such as chloride intracellular channel (CLIC)-dependent chloride (Cl^-^) efflux [30]. NLRP3 agonists induce K^+^ efflux, which causes mitochondrial damage and ROS production; in turn, mitochondrial ROS induce the translocation of CLICs to the plasma membrane for the induction of Cl^-^ efflux to promote inflammasome assembly and IL-1β secretion [31].

In addition to canonical NLRP3 inflammasome activation, a noncanonical inflammasome activated by LPS-induced caspase-11 has also been discovered. Caspase-11 activated by LPS interacts with its substrate, the protein GSDMD, and thus mediates noncanonical inflammasome activation [7].

NLRP3 activation is regulated by different proteins, such as never in mitosis A (NIMA)-related kinase 7 (NEK7), guanylate-binding protein 5 (GBP5) and double-stranded RNA-dependent protein kinase (PKR, also known as EIF2AK2) [32,33,34]. Lu and colleagues demonstrated for the first time that PKR physically interacts with inflammasome components, including NLRP3, NLRP1, NLRC4, and AIM2, and broadly regulates inflammasome activation [32]. Although a recent study suggested that PKR modulates inflammation by regulating the expression of the NLRP3 inflammasome through the NF-κB pathway in periodontal diseases [35], further studies are required to clarify the role of PKR in NLRP3 inflammasome activation. GBP5 seems to have a similar behavior in inflammasome activation, promoting selective NLRP3 inflammasome responses to pathogenic live bacteria and soluble but not crystalline agents and double-stranded DNA [33]. However, the role of GBP5 needs further clarification.

Growing evidence has shown a key role for NEK7, one of the smallest members of the NRK family, which regulates mitotic progression and the DNA damage response. NEK7 consists of a core kinase domain and a short N-terminal tail. Although the kinase domains of NEK6 and NEK7 are notably conserved, with 87% sequence identity, it has been demonstrated that NEK6 cannot control NLRP3 inflammasome activation like NEK7 can, suggesting the specificity of the NEK7-NLRP3 interaction [36].

In support of the idea that NEK7 is a selective upstream regulator of NLRP3 inflammasome activation, it has been demonstrated that when NEK7 is absent, the activation of caspase-1 and IL-1β is abrogated after stimulation with NLRP3 inflammasome activators, while this phenomenon is not observed for the NLRC4 or AIM2 inflammasome [37].

Under resting conditions, NEK7 activity is low, but any disruption of homeostasis may be accompanied by the dysregulation of NEK7. Emerging evidence has revealed that NEK7 upregulation is caused by a subunit of NF-κB, p65. p65 binds to the NEK7 promoter region via effects of the LPS-induced TLR4/NF-κB pathway and upregulates NEK7 expression [38]. Several studies have investigated the interaction between NLRP3 and NEK7. Previous studies have revealed that both the NACHT and LRR domains of NLRP3 are involved in the interaction with NEK7. Further works suggested that the N-terminal region of the NEK7 catalytic domain mediates the NLRP3-NEK7 interaction [39].

However, despite being a widely studied area of research, many parts of this complex mechanism are unclear. Clearly understanding the specific mechanisms of NLRP3 inflammasome activation could help broaden the field of NLRP3 inflammasome regulation and provide more potential therapeutic targets.

## 3. Role of the NLRP3 Inflammasome in Cancer

In this section, we will provide a general overview of the involvement of the NLRP3 inflammasome in tumorigenesis (Table 1).

### 3.1. Role of NLRP3 in Tumors of the Gastrointestinal Tract

#### 3.1.1. Colon Cancer

Colorectal cancer (CRC) is the third most common malignancy worldwide and has a high mortality rate due to its typical rapid progression with diagnosis at an advanced stage [99]. A Western diet, the aging of the world population and chronic inflammation are the main factors related to bowel inflammation and colitis-associated cancer (CAC). These factors lead to constant overproduction of pro-inflammatory cytokines such as IL-1β, IL-18, IL-6, TNF-α and DNA-damaging reactive oxygen and nitrogen species. All these molecules act as PAMPs and DAMPs that activate inflammasomes and promote intestinal epithelial cell proliferation, survival and angiogenesis, leading to epithelial dysplasia and invasive tumor formation [100].

The NLRP3 inflammasome and its activation in intestinal pathologies have been investigated predominantly in murine models of chemically induced intestinal inflammation. Such models feature epithelial barrier damage that is induced by invasion of gut microflora into the lamina propria and massive infiltration of inflammatory cells and upregulation of proinflammatory cytokines, which are features that are comparable to those seen in human ulcerative colitis [101]. However, the several studies that have been carried out in the last ten years depict a controversial role of NLRP3 in CRC, with some studies showing a protective role while others demonstrate a negative effect of NLRP3 activation.

The protective role of NLRP3 was suggested by experiments that showed that azoxymethane-dextran sodium sulfate (AOM-DSS) mice deficient in inflammasome components, including *Nlrp3*, *Asc*, *Caspase-1*, *Caspase-11*, *IL-18* and *IL-18r*, were more susceptible to CRC than those without deficiencies and exhibited accelerated tumor growth accompanied by attenuated levels of IL-1β and IL-18 [40,41,42,43]. Moreover, NLRP3 inflammasome-derived IL-1β and IL-18 play a protective role against oxazolone-induced colitis [44], and a hyperactive NLRP3 inflammasome (such as that in the *Nlrp3* R258W mutant mouse line) confers strong resistance to experimental colitis/CRC [45]. Intriguingly, the NLRP3 inflammasome suppresses CRC metastatic growth through IL-18 production by promoting hepatic natural killer (NK) cell tumoricidal activity [46]. In line with these published reports, Hu and colleagues reported data from a model in which caspase-1 deficiency enhanced inflammation-induced CRC formation through regulation of the epithelial cell response to injury; however, these effects were mediated through the NLRC4 inflammasome rather than through NLRP3 [47].

In contrast with these findings, another study reported that the NLRP3 inflammasome is a critical regulator of intestinal inflammation in the DSS colitis model [55], and *Nlrp3*^-/-^ mice develop less severe colitis than wild-type mice and produce lower levels of pro-inflammatory cytokines in colonic tissue [55]. Interestingly, administration of the small molecule andrographolide (Andro), a natural compound, prevents tumorigenesis in a colitis-associated CRC model by inducing mitophagy, which results in NLRP3 inflammasome inhibition [102].

However, the inconsistent observations between these studies comparing wild-type mice and *Nlrp3^-/-^* mice may stem from methodological differences between the experimental models (DSS or AOM-DSS) as well as from differences in the composition of the intestinal flora of the mouse lines used.

A crucial study found that NLRP3 expression was upregulated in human CRC tissues compared to adjacent normal tissues and was associated with tumor invasion and poor prognosis [56]. The NLRP3 signaling pathway might correlate with the mTOR-S6K1-MAPK signaling pathway, which synergistically promotes the invasion and migration of CRC cells [103]. This notion was supported by a genetic study that reported an association between single nucleotide polymorphisms (SNPs) in the *Nlrp3* gene and CRC patient survival, with NLRP3 SNPs contributing to an increase in IL-1β and subsequent IL-6 levels and a poor outcome [57]. Other studies determined that NLRP3 upregulation could contribute to CRC cell migration and invasion [58] via an inflammasome-independent mechanism [104].

#### 3.1.2. Pancreatic Cancer

Pancreatic ductal adenocarcinoma (PDA) is one of the most aggressive solid malignancies and has a devastating prognosis and limited therapeutic options. IL-1β is frequently upregulated in patients with pancreatic cancer (PC) and is associated with poor prognosis [62,63]. High NLRP3 signaling has been found in subsets of PDA-associated macrophages in both humans and mice, in which it promotes accelerated progression of PC [59]; moreover, increased NLRP3 expression correlates with proliferation and epithelial-mesenchymal transition (EMT)-induced cancer cell invasion [60]. Interestingly, the long noncoding RNA XLOC_000647 acts as a tumor suppressor and suppresses the progression of PC by downregulating NLRP3 [60].

IL-1β increases tumor infiltration of immunosuppressive macrophages and myeloid-derived suppressor cells (MDSCs), thereby promoting immune evasion, neoangiogenesis and tumor development [105]. IL-1β neutralization promotes intratumoral CD8^+^ T cell infiltration and function and sensitizes PDA to immunotherapy, confirming that the effects of tumor cell-derived IL-1β are NLRP3-dependent and identifying a tumor-supportive role for NLRP3 in PC [105]. In line with this notion, PC cell lines and tumor cell-conditioned macrophages are able to activate ASC and to induce the release of IL-1α and IL-1β which are crucial for the secretion of thymic stromal lymphoprotein (TSLP) by cancer-associated fibroblasts (CAFs), promoting Th2 inflammation [106] and increasing NF-kappaB activity and survival [107]. Treatment with anakinra, an IL-1R antagonist, in an orthotopic mouse model induced a reduction in TSLP expression with a downregulation of Th2 immunity, resulting in improved overall survival [106]. Intriguingly, the NLRP3 inflammasome can also control platelet activation, a key feature in PDA, by promoting platelet aggregation and cancer progression and interfering with patient survival [61]. Pharmacological inhibition of NLRP3 with MCC950 significantly inhibits platelet activation and aggregation and tumor progression [61].

Collectively, these data suggest that NLRP3 signaling accelerates the progression of pancreatic neoplasias and that targeting NLRP3 is a promising therapeutic strategy.

#### 3.1.3. Gastric Cancer

Gastric cancer (GC) is a common malignancy in the digestive system. Most of the evidence regarding GC and the NLRP3 inflammasome is derived from studies involving *Helicobacter pylori* (*H. pylori*): gastric infection with *H. pylori* is one of the most relevant factors implicated in GC development. *H. pylori* appears to activate the NLRP3 inflammasome [108,109], leading to IL-1β and IL-18 secretion [110,111], which regulates gastric immunity. Following *H. pylori* infection, NLRP3 expression is dramatically enhanced, and IL-1β secretion by macrophages is increased [64]. MiR-22 acts as a suppressor of NLRP3 expression in both gastric epithelial cells and macrophages and attenuates *H. pylori*-induced gastric carcinogenesis [64]. The mucin MUC1, known for its functions as an epithelial barrier, protects against *H. pylori*-induced gastritis by inhibiting NLRP3 activation [112]. Therefore, elimination of *H. pylori* infection seems significant for preventing GC; by contrast, *H. pylori* seems to prevent the critical activation of NLRP3 in human immune cells [113].

In addition to *H. pylori*, *Mycoplasma hyorhinis* also induces IL-1β secretion in an NLRP3-dependent manner both in vitro and in vivo, and it appears capable of promoting GC cell migration and invasion depending on inflammasome activation [65]. Notably, IL-1β-mediated inflammation has been linked to gastric carcinogenesis [66,67,68], and polymorphisms in the IL-1β and IL-18 genes that lead to an increase in the levels of these pro-inflammatory factors increase the susceptibility to the development of GC [114,115,116,117].

In conclusion, NLRP3 seems to have a definite role in promoting gastric carcinogenesis, but its role in other gastrointestinal malignancies is still debated.

#### 3.1.4. Hepatic Cancer

Hepatocellular carcinoma (HCC) is one of the most prevalent malignant tumors worldwide. Studies that have directly assessed the intrinsic role of NLRP3 in the development and progression of HCC have depicted a complex scenario in which its role appears to be twofold.

The expression of all NLRP3 components is significantly downregulated in human HCC, and this deficiency correlates with advanced stage and poor pathological differentiation [48]. Moreover, 17b-estradiol (E2) and estrogen receptor beta (ERβ) protect against HCC by activating the NLRP3 inflammasome [118] and by triggering pyroptotic cell death and inhibiting protective autophagy [119,120]. Interestingly, NLRP3 is required for the suppression of CRC metastatic growth in the liver, and its tumor-suppressive function is mediated by IL-18 production, which acts on NK cells [46].

In sharp contrast with these notions, there are several studies that support protumoral activity of NLRP3 in the pathogenesis of HCC. Luteoloside, a natural flavonoid with several pharmacological activities, reduces intracellular ROS accumulation and thus suppresses NLRP3 inflammasome activation, limiting the proliferation and metastasis of HCC cells [121]. In line with this, the NLRP3 inflammasome pathway has been described to have a procarcinogenic effect in hep3B cells, in which tumor-suppressive miR-223 promotes apoptosis and inhibits the proliferation of HCC cells by negatively regulating NLRP3 and downstream cytokines [122]. Mice treated with diethylnitrosamine (DEN), a potent liver carcinogen, display elevated levels of NLRP3 and IL-1β, which have been associated with inflammatory conditions. Combination therapy with celastrol and metformin suppresses NLRP3 and IL-1β, enhances anti-inflammatory and antioxidant activities, and downregulates angiogenesis, metastasis and proliferation markers [123]. Similarly, anisonamide suppresses the growth of HCC and induces apoptosis by inhibiting NLRP3 expression and further inhibiting the activation of the NLRP3 inflammasome, thus reducing the production of IL-1β [124]. Recently, Brocker and colleagues provided evidence for a mechanism by which proliferator-activated receptor α (PPARα) suppresses hepatic inflammation during metabolic stress by targeting the lncRNA *Gm15441* [125]. *Gm15441* suppressed TXNIP and thus TXNIP-NLRP3 inflammasome activation, caspase-1 cleavage and the expression of IL-1β [125]. The authors speculate that *Gm15441* is a plausible therapeutic target for the treatment of inflammatory disorders, potentially including HCC [125].

Taken together, these results unveil a dual role for the NLRP3 inflammasome in the pathogenesis of HCC, and additional studies are essential to deeply understand the relationship between HCC progression and the NLRP3 inflammasome.

### 3.2. Involvement of NLRP3 in Gynecological Cancers

#### 3.2.1. Endometrial Carcinoma

Among gynecological cancers, endometrial carcinoma is the most common in developed countries, and its incidence is increasing [126]. Studies on inflammasome complexes have strengthened the correlations between estrogenic stimulation, inflammation and the development of endometrial neoplasia. Recently, the expression of NLRP3, its inflammasome components and ERβ was analyzed in endometrial cancer tissue samples from 31 patients [70]. Molecular analysis showed that estrogens enhanced the proliferation of endometrial cancer cells by upregulating NLRP3 expression via ERβ. On the other hand, knockdown of NLRP3 expression inhibited the growth of cancer cells and reduced caspase-1 activation and IL1β maturation. Therefore, the different roles of estrogen/ERβ/NLRP3 inflammasome activation may be related to estrogen levels and ERβ expression in endometrial cancer patients [70].

Moreover, Yang et al. confirmed the hyperexpression of NLRP3 and related proteins in endometrial cancer in an attempt to link them with the pyroptosis pathway [71].

#### 3.2.2. Cervical Cancer

Cervical cancer is the second most common neoplasia in females worldwide. Its genesis is related to high-risk human papillomavirus (HR-HPV) infection [127]. However, HPV itself seems to be insufficient to induce the malignant transformation process. Chronic inflammation, along with HPV infection, is involved in carcinogenesis [128,129]. In 2016, Pontillo et al. investigated 12 SNPs in 7 inflammasome-related genes as possible risk factors for HPV infection susceptibility and/or progression to cervical cancer [72]. They found a statistically significant association between the *Nlrp3* variant rs10754558 and HR-HPV resistance. Their results demonstrate that inflammasome genetics can affect HPV/host interactions in terms of virus susceptibility, virus persistence and cervical cancer progression [72]. Furthermore, the membrane glycoprotein CD200 of the immunoglobulin superfamily, and its soluble formulation CD200 fusion protein Fc (CD200Fc), has been demonstrated to play an active role in the suppression of the inflammatory activity of the TLR4-NF-kappaB and NLRP3 inflammasome pathways in LPS-induced cervical cancer cell lines [130].

#### 3.2.3. Ovarian Cancer

Ovarian cancer is the third most common gynecological malignancy and has the highest mortality rate. Epithelial ovarian cancer (EOC) is the pathological subtype of more than 90% of cases [131].

Several factors including molecular pathway dysfunctions, such as deregulation of oxidoreductase activity, metabolism, hormone activity, the inflammatory response, the innate immune response, and cell–cell signaling have been involved in its pathogenesis. Only a few studies have so far investigated the role of NLRP3 in the pathogenesis of EOC. One study investigated the molecular mechanism involved in the malignant transformation of endometriosis nodules into clear cell and endometrioid EOC, analyzing a list of 18 genes related to the inflammasome complex, and indicated a role of inflammation/immunity in EOC transformation [73]. The study explored the correlation between these genes and patient survival, demonstrating a statistically significant correlation between high expression levels of NLRP3 and poor prognosis. These results suggest that inflammasome dysregulation could play a fundamental role in modulating malignant transformation of endometriosis and that NLRP3 signaling concomitant with persistent sterile inflammation could indicate the initial stage of ovarian carcinogenesis [73].

Luborsky et al. studied whether NLRP3 inflammasome components and their corresponding cytokine products were increased in ovarian tumors by analyzing chicken and human normal ovaries and ovarian tumors [132]. They found that the expression of caspase-1, IL-1β and IL-18 was higher in ovarian cancer tissues than in normal tissues; however, they found no difference in NLRP3 expression. The reasons why immune reactions may differ during the development and progression of ovarian tumors remain to be determined, but this study laid the foundation for future evaluation of the relationship between tumor initiation and early inflammatory events involved in ovarian cancer development [132]. Recently, the correlation between the survival of EOC patients and the expression levels of inflammasome components and their related genes has been investigated, emphasizing that high expression levels of NLRP3 are associated with poor patient prognosis [74].

All these results and some additional evidence [133] support the usage of NLRP3 inflammasome genes and their corresponding proteins as potential markers of EOC progression and prognosis and as potential therapeutic targets.

### 3.3. NLRP3 Inflammasome Involvement in Other Types of Cancer

The NLRP3 inflammasome complex has attracted considerable attention for its role in the development of several other cancer types, but this role remains unclear and controversial.

#### 3.3.1. Lung Cancer

Lung cancer (LC) is a major cause of death in industrialized countries. The most frequent form is non-small-cell lung cancer (NSCLC), representing 85% of cases [134]. Several studies have established the link between chronic inflammation and LC, but they depict a setting in which the involvement of the NLRP3 inflammasome is controversial [135]. Alveolar macrophages (AMs), critical in local lung inflammation, from patients with LC show downregulation of NLRP3/caspase-1 inflammasome activation, which is characterized mainly by the impairment of IL-1β production [49]. Conversely, tumor-derived exosomal TRIM59 has been shown to rewire macrophages towards a protumoral pathway by degrading ABHD5 protein and exacerbating NLRP3 inflammasome activation, which promotes LC cell proliferation and invasion [75].

Overexpression of IL-1β in lung adenocarcinoma (ADC) cells results in the promotion of experimental lung metastasis via enhanced expression of adhesion-, invasion- and angiogenesis-related molecules [76]. In line with this, NLRP3 promotes the mouse lung tumorigenesis induced by benzo(a)pyrene and LPS [77] and favors experimental lung metastasis development by affecting the ability of host NK cells to control the tumor [78]. Polydatin, a natural component, suppresses the proliferation and migration of NSCLC cells via the NF-κB pathway, inhibiting NLRP3 inflammasome activation [136,137]. In contrast, experimental metastasis is more pronounced in mice lacking IL-1β than in wild-type mice due to the increased number of regulatory T cells in mice lacking IL-1β, which favors the malignant process [138].

Interestingly, NLRP3 inflammasome elements were overexpressed in high-grade ADC, which is an NSCLC subtype, and in small-cell lung cancer (SCLC) [79], suggesting that they could be crucial biomarkers for LC as well as potential modulators of the biological behaviors of LC. Recently, the association between genetic variants in NLRP3 inflammasome genes and the survival of NSCLC patients has been assessed; two novel genetic variants (in *BIRC3* and *NRG1*) have been associated with NSCLC and may affect patient survival [139].

Altogether, these data correlate the NLRP3 inflammasome with LC development; however, the exact molecular mechanisms linking the NLRP3 inflammasome to LC development are still unclear, and further investigation is needed.

#### 3.3.2. Breast Cancer

Breast cancer (BC) is the leading cause of mortality in women, with BC accounting for 14–15% of all female cancer deaths worldwide [140].

Current studies underline the central role of the microenvironment and infiltrating immune cells in the migration and invasion of BC cells [141,142]. CAFs are an abundant population in the microenvironment of BC and take a center stage in tumorigenesis [143,144]. Recently, it has been proposed that CAFs sense DAMPs and thus activate the NLRP3 inflammasome pathway, leading to proinflammatory signaling and IL-1β release, which promote tumor progression and lung metastasis [144]. Targeting the NLRP3-IL-1β pathway activated by CAFs may be a beneficial approach for BC treatment. Accordingly, transcription factor EB (TFEB) modulates tumor-associated macrophage (TAM) gene expression in BC through several pathways, for example, by promoting NLRP3 inflammasome degradation [80]. Moreover, NLRP3 expression in tumor-infiltrating macrophages has been correlated with the survival, lymph node invasion and metastasis of mammary carcinoma patients [81]. Human BC tissues display unc-51-like autophagy activating kinase 1 (ULK1) deregulation that contributes to mitochondrial ROS accumulation and thus NLRP3 activation, which in turn promotes BC osteolytic bone metastasis [82].

In contrast with this view, Huang and colleagues demonstrated that NLRP3 activation is necessary to induce antitumor immunity in patients with BC [50]. Mechanistically, they identified that myeloid PTEN determines chemotherapy efficiency by activating the NLRP3 inflammasome, suggesting that manipulating the NLRP3-IL-1β axis might be able to overcome chemotherapy resistance in patients with tumors with PTEN loss [50].

The definitive functions of the NLRP3 inflammasome in mammary malignancy are still unknown, but several studies have demonstrated the pivotal function of IL-1β in tumorigenesis and neoplasm growth [145,146]. The inflammatory cytokine IL-1β positively regulates mammary tumor growth and invasiveness by affecting the nature of myeloid cells and promoting TAM differentiation [83]. In a transgenic mouse model, inducible fibroblast growth factor receptor 1 (iFGFR1) induces an increase in IL-1β that in turn regulates cyclooxygenase-2 expression, which promotes cell migration and favors mammary lesions [147].

Increased IL-1β levels in primary tissues and metastatic sites have been detected in a spontaneous murine mammary gland tumor model, suggesting that the IL-1β pathway promotes tumor growth and metastasis [84]. Significantly, IL-1R blockade with an antagonist reduces tumor growth and inhibits human BC progression [84].

Although further studies are needed, these data depict a clear protumoral role for NLRP3 in mammary cancer.

#### 3.3.3. Head and Neck Squamous Cell Carcinoma

NLRP3 inflammasome activation has been demonstrated to promote inflammation-induced carcinogenesis in head and neck squamous cell carcinoma (HNSCC) [85], and P2 × 7R and NLRP3 have been found to be overexpressed in HNSCC in association with increased survival and invasiveness [86]. Additionally, the IL-1β concentration was increased in the peripheral blood of HNSCC patients [87]. In contrast, cluster of differentiation 38 (CD38) has been demonstrated to act as a tumor suppressor gene in HNSCC, triggering pyroptotic cell death by activating NLRP3 and caspase-1 [51]; thus, by inhibiting CD38 expression, HNSCC cells might be able to escape NLRP3 inflammasome-mediated pyroptosis [51].

#### 3.3.4. Oral Cavity Squamous Cell Carcinoma

Interestingly, the levels of the NLRP3 complex components (NLRP3, ASC and IL-1β) were significantly elevated in oral cavity squamous cell carcinoma (OSCC) and contributed to tumor growth and metastasis [88,89]. High NLRP3 expression has been associated with a more malignant phenotype in OSCC and promotes 5-fluorouracil (5-FU) resistance [148]; therefore, targeting the ROS/NLRP3 inflammasome pathway may decrease the resistance of OSCC cells to 5-FU-based therapy [148]. As already demonstrated for gastric cancer, miR-22 suppresses growth, migration and invasion in OSCC cells by affecting the functions of the NLRP3 inflammasome [149]. Taken together, these data suggest that the NLRP3 inflammasome may be a potential target for OSCC therapy.

#### 3.3.5. Skin Cancers

By performing a pancancer analysis of NLRP3 inflammasome-related genes across 24 human cancers, Ju M. and colleagues found that 15 cancers had significant differences in NLRP3 expression between normal and tumor samples [150]. Intriguingly, the researchers showed that highly malignant HCC and skin cutaneous melanoma (SKCM) were associated with low NLRP3 inflammasome scores, which could serve as independent prognostic factors in SKCM [150]. The mechanisms of the NLRP3 inflammasome in melanoma and other skin cancers are not clearly understood [151], but several reports have demonstrated the involvement of NLRP3 inflammasome components in tumorigenesis. Polymorphisms of NLRP3 are associated with the development of melanoma [90], and *IL-1R^-/-^* and *caspase 1^-/-^* mice rarely develop skin cancer after stimulation with chemical agents [91]. The NLRP3 inflammasome shows different levels of pathway activation according to disease stage: it is constitutively activated in late stages of melanoma, producing high levels of IL-1β in the microenvironment [152,153], while IL-1β secretion has been found to require the activation of the receptor IL-1R during intermediate stage cancer in vitro [153]. Moreover, Drexler et al. showed that infiltrating myeloid cells were mainly responsible for tumor-promoting IL-1β release in the tumor microenvironment [91]. Interestingly, the adaptor protein ASC also plays opposite roles in skin tumorigenesis, acting as a driver of tumorigenesis when expressed in infiltrating myeloid cells but as a tumor suppressor when expressed in keratinocytes [91].

#### 3.3.6. Prostate Cancer

It is known that the microenvironment plays a pivotal role in both cancer development and progression [154]. IL-1β abundance in the tumor environment and its overexpression have already been associated with the progression of different cancer types [69], including prostate cancer (PCa) [93,155]. High levels of IL-1β and IL-18 have been found in the serum of patients with advanced PCa [92,156]. The proinflammatory cytokine IL-1β is overexpressed in an in vitro model of PCa, suggesting the activation of the NLRP3/caspase-1 pathway in malignant growth [93]. Cancer cell-derived extracellular vesicles (EVs) can modify the prostate microenvironment to promote cancer proliferation [157] and can promote the activation of the NLRP3 inflammasome and caspase-1 and IL-1β release, contributing to the maintenance of proinflammatory tumor conditions [93]. However, immunohistochemical experiments in human prostate samples demonstrated that there were no significant differences in NLRP3 expression between tumor and adjacent tissues, either benign or malignant, suggesting that NLRP3 might not be involved in PCa metastasis [158].

#### 3.3.7. Gliomas

The role of the NLRP3 inflammasome and its components has also been extensively described in gliomas, a group of malignant neoplasms of the central nervous system, which led to the identification of a protumoral role for NLRP3 [159]. The upregulation of NLRP3 affects human glioma progression by activating the AKT signaling pathway [94] and IL-1β/NF-kappaB p65 signaling [160], thus promoting migration and invasion. In addition, glioma cells display aberrant expression of IL-1β, and NLRP3 seems to contribute to radiotherapy resistance [95]. Interestingly, beta-hydroxybutyrate has been recently reported to counteract cell migration by suppressing the activation of caspase-1 and the maturation of IL-1β [161].

#### 3.3.8. Hematological Malignancies

To date, studies have addressed the role of NLRP3 in solid tumors, but this inflammasome has an important and still controversial role in hematological malignancies. It has been demonstrated that patients with primary lymphoma and acute myeloid leukemia (AML) display markedly increased expression of NLRP3 inflammasome molecules accompanied by overexpression of IL-18 mRNA [96,98]; nevertheless, chronic myeloid leukemia (CML) patients display lower levels of IL-1β or NLRP3 than controls [52]. Oncogenic KRAS leads to ROS production, NLRP3 activation and consecutive release of IL-1β in a myeloid leukemia mouse model, and KRAS-mutant human leukemia cells exhibit increased NLRP3 inflammasome activation [97]. Moreover, the activation of the NLRP3 inflammasome reduces the antitumor effects of dexamethasone in lymphoma, altering the c-myc/TP53 and bcl-2/bax equilibrium [98].

In contrast, the NLRP3 inflammasome seems to have antitumor activity in multiple myeloma (MM) [53] and chronic lymphocytic leukemia (CLL) [54], in which it is expressed at lower levels in cancer patients than in healthy controls. The downregulation of NLRP3 in CLL lymphocytes blocks cell proliferation and induces apoptosis, suggesting that the NLRP3 inflammasome works as a negative regulator of tumor growth [54].

**Figure 2:**
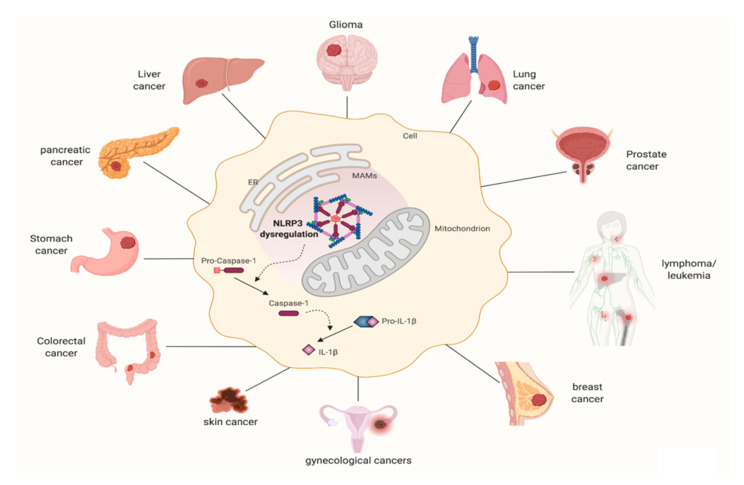
NLRP3 inflammasome involvement in distinct types of cancer.

Taken together, these data suggest that a unified mechanism for NLRP3 inflammasome activation and its involvement in cancer has not yet emerged, so future studies are needed to better clarify its role in each kind of tumor (Figure 2).

## 4. NLRP3 Inflammasome as a Promising Target for Cancer Therapy

The clinical relevance of the NLRP3 inflammasome in multiple forms of cancer highlights its therapeutic promise as a molecular target. Here, we list the several compounds that impinge on inflammasome activation or inhibit downstream products of inflammasome activation and that can affect cancer development (Table 2).

The first inhibitor of NLRP3 to be discovered was the sulfonylurea CP-456773/CRID3, recently named MCC950, a compound found to be a potent and specific small-molecule inhibitor of the NLRP3 pathway [162]. From a molecular point of view, MCC950 directly interacts with and affects the Walker B motif within the NLRP3 NACHT domain, thereby blocking ATP hydrolysis [163] and inducing NLRP3 to transition into an inactive conformation [164]. To date, MCC950 has been tested in more than 80 animal models of more than 50 diseases, and these studies triggered a major increase in the amount of NLRP3 research. In cancer treatment, MCC950 was found to suppress and delay tumor growth and reshape the antitumor response in an HNSCC mouse model by decreasing the number of immunosuppressive cells and improving T cell functions [87]; moreover, MCC950 was able to reduce cell proliferation in a panel of PC cells by inhibiting inflammation [165]. Notably, MCC950 reverses KRAS-driven cytopenia and myeloproliferation [97]. MCC950 was shown to be effective in inhibiting IL-1β secretion and activation of caspase-1 in murine ulcerative colitis [166]. Other compounds have been shown to be effective for the treatment of DSS-induced experimental colitis in mice. A novel tetrahydroquinoline inhibitor of NLRP3, compound 6, specifically inhibits NLRP3 activation in vivo and attenuates colitis severity in the DSS mouse model [167] by directly binding to the NACHT domain of NLRP3, inhibiting its ATPase activity and blocking ASC oligomerization [167]. 1-Ethyl-5-methyl-2-phenyl-1H-benzo[d]imidazole, a synthetic small molecular compound also named Fc11a-2, was shown to induce beneficial effects in the treatment of DSS-induced experimental colitis in mice by targeting the NLRP3 inflammasome and inhibiting cytokine release [168]. VI-16, a synthetic flavonoid compound, exerts potent anti-inflammatory effects on macrophages by inhibiting the binding of TXNIP to NLRP3 and thus reducing ROS production and inhibiting the NLRP3 inflammasome [169]. NF-kappaB signaling and consequent NLRP3 inflammasome activation have been shown to be suppressed by fraxinellone, a lactone compound [170], and by alpinetin, a novel plant flavonoid [171]; both alleviate DSS-induced colitis.

Recently, the omega class glutathione transferase (GSTO1-1) inhibitor C1-27 has been demonstrated to regulate the release of IL-1β and IL-18 by deglutathionylating NEK7 in the NLRP3 inflammasome [172] and thus to protect against CRC formation [173].

These studies provide evidence that NLRP3 inflammasome inhibitors can function as potential novel therapeutic agents for human inflammatory bowel disease and, considering the correlation between the NLRP3 inflammasome and cancer, suggest new avenues for CAC treatment.

Celastrol, a natural triterpene, has been demonstrated to be an important inhibitor of the NF-kappaB signaling pathway [174] and of the NLRP3 inflammasome, and suppressing caspase-1 activation and IL-1β secretion prevents DSS-induced colitis [175] and reduces the ability of macrophages to stimulate the migration and invasion of melanoma cells [152].

Oridonin specifically inhibits NLRP3 by forming a covalent bond with cysteine 279 of the NLRP3 NACHT domain to block the interaction with NEK7, thereby inhibiting NLRP3 inflammasome assembly [176]. Although there is no direct evidence that oridonin induces anticancer activity by inhibiting the NLRP3 inflammasome, its ability to suppress cell proliferation, migration and invasion has been described in ovarian [177], breast [179], osteosarcoma [178] and esophageal [180] cancers. The same idea may be applied to thalidomide, an efficient anti-inflammatory drug that is able to inhibit caspase-1 activation [181] and has antitumor activity in MM and PCa treatment [182,183].

Among small-molecule compounds with NLRP3 inhibitory activity, CY-09 increases gemcitabine sensitivity in triple-negative BC, limiting the activation of the EMT/IL-1β/Wnt/β-catenin signaling pathway [185]. CY-09 specifically binds the ATP-binding motif of NLRP3 and inhibits its ATPase activity [184].

The acetylase inhibitor SI-2, usually used as an antitumor reagent for anaplastic thyroid carcinoma 31977311, specifically inhibits NLRP3 inflammasome activation by disrupting the interaction between NLRP3 and ASC and by blocking ASC speck formation [186].

Despite the numerous molecules and drugs that have been shown to regulate inflammasome activity, the current treatments for NLRP3-related diseases in the clinic involve targeting IL-1β or IL-18. Canakinumab, a human anti-IL1β monoclonal antibody developed by Novartis, has evident antitumor effects against NSCLC [189,190] and is currently being tested in clinical trials for triple-negative BC, CRC, metastatic melanoma and PC treatment. Anakinra is a recombinant IL-1 receptor antagonist (IL-1RA), a well-known antioxidant and anti-inflammatory agent, that is able to block the signaling of both IL-1α and IL-1β through IL-1R [191]. Recently, anakinra has been demonstrated to mitigate glioblastoma aggressiveness by inhibiting the expression of proinflammatory cytokines and STAT3 [192], and as already mentioned, MCC950 is able to reverse KRAS-driven cytopenia and myeloproliferation [97]. Interestingly, anakinra significantly reduces the development and progression of BC bone metastasis [193]. Anakinra has been used in combination with other drugs in clinical trials: (i) with 5-FU and bevacizumab, in which it improved the survival rate and overall survival of patients affected by CRC [194]; (ii) with gemcitabine, nab-paclitaxel and cisplatin, in which it improved clinical outcomes in PDA (NCT02550327); and iii) with dexamethasone for treating patients with MM, in which it improved survival benefits. (NCT00635154).

Taken together, these findings highlight that directly targeting the NLRP3 inflammasome or its downstream pathways has begun to attract attention as a potential strategy for the development of novel anticancer therapies.

## 5. Concluding Remarks

Several biotech and pharmaceutical companies are now enthusiastically developing NLRP3 inhibitors that they think could address a wide variety of common diseases. Initially, most inhibitors of NLRP3 were developed to block the cytokines produced by its activation; at present, development strategies focus on compounds that directly bind to and perturb NLRP3 or target proteins that regulate posttranslational modification or degradation of inflammasome proteins. However, studies that have directly assessed the intrinsic role of NLRP3 in the regulation of cancer have depicted a complex scenario that mainly involves its aberrant expression or function. The seemingly contradictory function of inflammasome-dependent cytokines in tumor promotion and antitumor immunity is likely a result of the context-dependent and tissue-specific nature of such cytokines. Thus, to translate anti-NLRP3-based anticancer agents from the bench to the bedside, it will be important not only to identify molecules that selectively target NLRP3 or its downstream pathways in malignant cells but also to consider the metabolic heterogeneity of these malignant cells and the mechanisms through which such heterogeneity is connected to cancer.

In conclusion, alterations in NLRP3 inflammasome activation influence malignant transformation, tumor progression, and response to therapy by affecting an intricate network of cancer cell functions. Additional studies to disentangle the molecular and functional complexity of this network are urgently awaited.

Schematic representation of the several cancer types in which NLRP3 inflammasome is involved. Created with BioRender.com.

## Figures and Tables

**Table 1:** Role of NLRP3 inflammasome in cancer.

Outcome	Type of Cancer	Experimental Models	Impact on Cancer	References
anti tumoral role	Colon cancer	*Nlrp3*^-/-^, *Asc^-^*^/-^ and *Caspase1^-^*^/-^ mice	Increased DSS-induced colitis inflammation and tumorigenesis	[40,41,42,43]
		*Nlrp3*^-/-^ and *Caspase1*^-/-^ mice	Increased oxazolone-induced colitis	[44]
		*Nlrp3*^R258W^ mutant mice	Resist induced colitis and colorectal cancer	[45]
		*Ice*^−/−^ and *Nlrp3*^-/-^ mice	Enhanced colorectal cancer growth	[46]
		*Caspase1*^-/-^ mice	Enhanced AOM-DSS colitis CAC model	[47]
	Hepatic cancer	HCC and adjacent normal tissue	Loss of the NLRP3 is correlated with a Higher HCC Pathological Grade	[48]
		*Nlrp3*^-/-^and *Caspase1*^-/-^ mice	Highly susceptible to CRC liver metastatic growth	[46]
	Lung cancer	cells from LC patients and controls	NLRP3 and caspase 1 levels were decreased in LC cells	[49]
	Breast cancer	*Pten*^-/-^ mice	NLRP3 deficiency promotes chemotherapy resistance	[50]
	HNSCC	HNSCC cell lines	CD38 triggers NLRP3 inflammasome-mediated pyroptotic cell death	[51]
	Chronic myeloid leukemia	CML patients and controls	Lower levels of IL-1β and NLRP3 in CML patients compared to controls	[52]
	Multiple myeloma	cells from MM patients and controls	Downregulation of NLRP3 inflammasome in patients with MM compared to controls	[53]
	Chronic lymphocytic leukemia	cells from CLL patients and controls	Downregulation of NLRP3 inflammasome in patients with CLL compared to controls	[54]
pro-tumoral role	Colon cancer	*Nlrp3*^-/-^, *Asc*^-/-^ and *Caspase1*^-/-^ mice	Protected from DSS-colitis model	[55]
		CRC tissue	NLRP3-positive patients had a poor prognosis.	[56]
		CRC and adjacent normal tissue	Polymorphisms in NLRP3 are associated with poor survival	[57]
		macrophages surrounded CRC tissue	NLRP3 activation increased migration of CRC	[58]
	Pancreatic cancer	KC; *Nlrp3*^-/-^, *Asc*^-/-^ and *Caspase1*^-/-^ mice	Protected from pancreatic ductal adenocarcinoma	[59]
		PC and adjacent normal tissue	NLRP3 promotes proliferation, invasion of PC cells	[60]
		*Nlrp3*^-/-^ mice	The platelet NLRP3 promotes platelet aggregation and tumor growth	[61]
		pancreatic cyst fluid	high IL-1β levels in patients with high-grade dysplasia or cancer	[62,63]
	Gastric cancer	GC and adjacent normal tissue	Enhanced NLRP3 level correlates with GC progression	[64]
		GC cells	IL-1β promotes gastric cancer cell migration and invasion.	[65,66,67,68,69]
	Hepatic cancer	HCC cells	Downregulation of NLRP3, caspase-1, IL-1β and IL-18 favor apoptosis of the HCC cell	[48]
	Endometrial carcinoma	Endometrial cancer and adjacent normal tissue	NLRP3, ASC, caspase-1, and IL-1β upregulation promote cancer progression and poor survival.	[70]
		Endometrial cancer and adjacent normal tissue	Overexpression of NLRP3 and caspase-1 in human endometrial cancer	[71]
	Cervical cancer	cervical cancer tissue HPV+ or HPV-	NLRP3 SNPs are associated to progression to cervical cancer	[72]
	Epithelial Ovarian Cancer	EOC and adjacent normal tissue	Enhanced NLRP3 levels correlate with EOC progression	[73,74]
	Lung cancer	exosomes of LC cells	NLRP3 inflammasome activation promote LC progression by IL-1β secretion	[75]
		human LC cell lines	Enhanced IL-1β levels correlate with tumor cells proliferation and metastasis	[76]
		*Nlrp3*^-/-^ mice	NLRP3 deletion inhibits inflammation-driven mouse lung tumorigenesis	[77]
		*Nlrp3*^-/-^ mice	NLRP3 activation promotes lung metastasis	[78]
		LC and adjacent normal tissue	Enhanced NLRP3 levels in ADC and SCLC	[79]
	Breast cancer	human breast CAFs	NLRP3 facilitate tumor progression and metastases	[80]
		BC and adjacent normal tissue	NLRP3 expression in macrophages correlates with survival and metastasis	[81]
		BC and adjacent normal tissue	NLRP3 activation promotes BC bones metastasis	[82]
		*IL-1β*^-/-^ mice	IL-1β regulates mammary tumor growth and invasiveness.	[83]
		*Nlrp3*^-/-^and *IL-1R*α mice	NLRP3 and IL-1β promote tumor growth and metastasis	[84]
	HNSCC	HNSCC and adjacent normal tissue	NLRP3 inflammasome is upregulated and associated with carcinogenesis	[85,86,87]
	OSCC	OSCC cell lines and tissue	Enhanced NLRP3, ASC and IL-1β levels correlate with tumor growth and metasases	[88,89]
	melanoma	melanoma and adjacent normal tissue	NLRP3 polymorphisms is associated with melanoma susceptibility	[90]
		*IL-1R1*^-/-^ and *Caspase-1*^-/-^	Inflammasome-dependent production of IL-1β favors tumorigenesis.	[91]
	Prostate cancer	Pca cell lines and tissue	Enhanced IL-18 correlate with tumor status	[92]
		Pca cell lines	Enhanced IL-1β correlate with tumor stage	[93]
	Glioma	glioma cell lines and tissue	Enhanced NLRP3, ASC, caspase-1 and IL-1β levels correlate with with higher glioma grades; NLRP3 promotes glioma cell migration and invasion	[94]
		glioma cell lines	NLRP3 inflammasome contributed to radiotherapy resistance in glioma.	[95]
	Acute myeloide leukemia	cells from AML patients and controls	Enhanced NLRP3 levels in patients with AML	[96]
		KrasG12D; *Nlrp3*^-/-^ mice and human leukemia cells	NLRP3 enhances myeloproliferation and cytopenia	[97]
	Lymphoma	lymphoma patients and control	Polymorphism of IL-18 (rs1946518) was significantly associated with lymphoma susceptibility	[98]

Abbreviations: AML: acute myeloid leukemia; AOM: azoxymethane; BC: breast cancer; CAFs: cancer associated fibroblasts; CLL: chronic lymphocytic leukemia; CML: chronic myeloid leukemia; CRC: colorectal cancer; DSS: dextran sodium sulphate; GC: gastric cancer; HCC: hepatocellular carcinoma; HNSCC: head and neck squamous cell carcinoma; HPV: human papillomavirus; LC: lung cancer; MM: multiple myeloma; OSCC: oral cavity squamous cell carcinoma; PC: pancreatic cancer; PCa: prostate cancer.

**Table 2:** List of compounds that impinge on NLRP3 inflammasome activation and have a potential in cancer treatment.

Compound Name	Target	References	Effective Cancer Type	References
MCC950	directly interacts with the Walker B motif within the NLRP3 NACHT domain, thereby blocking ATP hydrolysis and its active conformation	[162,163,164]	HNSCC	[87]
			PCa	[165]
			CML	[97]
			PDA	[61]
			DSS-induced experimental colitis in mice	[166]
Compound **6**	directly binds the NLRP3 NACHT domain, thereby blocking ATPase activity and ASC oligomerization	[167]	DSS-induced experimental colitis in mice	[167]
Fc11a-2	targets NLRP3 inflammasome and inhibits cytokines release	[168]	DSS-induced experimental colitis in mice	[168]
VI-16	inhibits the binding of TXNIP to NLRP3 by reducing NLRP3 activation	[169]	DSS-induced experimental colitis in mice	[169]
Polydatin	suppresses NF-kappaB signaling and NLRP3 inflammasome activation	[136]	NSCLC	[137]
Fraxinellone	suppresses NF-kappaB signaling and NLRP3 inflammasome activation	[170]	DSS-induced experimental colitis in mice	[170]
Alpinetin	inhibits NF-kappaB pathway and NLRP3 inflammasome activation	[171]	DSS-induced experimental colitis in mice	[171]
C1-27	limits NLRP3 activation by reducing ASC speck formation	[172]	CRC	[173]
Celastrol	inhibits NF-kappaB pathway, blocks ASC oligomerization and NLRP3 complex formation	[174,175]	DSS-induced experimental colitis in mice	[175]
			melanoma cancer cells	[152]
Oridonin	blocks NEK7-NLRP3 interaction by binding the cysteine 279 on NLRP3 NACHT domain	[176]	ovarian cancer	[177]
			osteosarcoma	[178]
			BC	[179]
			esophageal cancer	[180]
Thalidomide	inhibits caspase-1 activation	[181]	MM	[182]
			PCa	[183]
CY-09	binds to the ATP-binding motif of NLRP3 NACHT domain and inhibits NLRP3 ATPase activity.	[184]	triple negative BC	[185]
SI-2	disrupts the interaction between NLRP3 and ASC then blocks the formation of ASC speck	[186]	anaplastic thyroid carcinoma	[187]
Andrographolide	inhibits caspase-1 activation	[102]	DSS-induced experimental colitis in mice	[102]
Canakinumab	IL-1β inhibitor	[188]	NSCLC	[189,190]
			triple negative BC, CRC, metastatic melanoma and PC	-
Anakinra	IL-1 receptor antagonist	[191]	glioblastoma	[192]
			AML	[97]
			BC	[193]
			CRC	[194]
			PDA	-
			MM	-

Abbreviations: AML: acute myeloid leukemia; BC: breast cancer; CAFs: cancer associated fibroblasts; CML: chronic myeloid leukemia; CRC: colorectal cancer; DSS: dextran sodium sulphate; LC: lung cancer; MM: multiple myeloma; PCa: prostate cancer; PDA: pancreatic ductal adenocarcinoma.

## Data Availability

Not applicable.

## Abbreviations

5-FU	5-fluorouracil;
ADC	adenocarcinoma;
AML	acute myeloid leukemia;
AOM	azoxymethane;
ASC	apoptosis-associated speck-like protein;
Andro	andrographolide;
ATP	adenosine triphosphate;
BC	breast cancer;
Ca^2+^	calcium ion;
CAC	colitis-associated cancer;
CAFs	cancer associated fibroblasts
CAPSs	cryopyrin-associated periodic syndromes;
CARD	caspase activation and recruitment domain;
CLICs	chloride intracellular channels;
CLL	chronic lymphocytic leukemia;
CML	chronic myeloid leukemia;
CRC	colorectal cancer;
DAMPs	damage-associated molecular patterns;
DEN	diethylnitrosamine;
DSS	dextran sodium sulphate;
EMT	epithelial-mesenchymal transition;
EOC	epithelial ovarian cancer;
ER	endoplasmic reticulum;
ERβ	Estrogen Receptor β;
EVs	extracellular vesicles;
FADD	FAS-associated death domain protein;
GBP5	guanylate-binding protein 5;
GC	gastric cancer;
GSDMD	gasdermin D;
GSTO1-1	omega class glutathione transferase;
HCC	hepatocellular carcinoma;
HNSCC	head and neck squamous cell carcinoma;
H. pylori	Helicobacter pylori;
HR-HPV	high risk human papillomavirus
IL-β	interleukin-1beta;
IL-18	interleukin-18;
IL-1RA	IL-1 receptor antagonist;
K^+^	potassium;
LC	lung cancer;
LRR	leucine-rich repeat;
LPS	lipopolysaccharide
MAMs	mitochondria-associated membranes;
MDSC	myeloid-derived-suppressor cells;
MM	multiple myeloma;
NACHT	nucleotide-binding and oligomerization;
NEK7	never in mitosis A (NIMA)-related kinase 7;
NF-kappaB	nuclear factor-kB
NK	natural killer;
NLRP3	NLR family protein containing a pyrin domain 3;
NSCLC	non-small-cell lung cancer;
OSCC	oral cavity squamous cell carcinoma;
PAMPs	pathogen-associated molecular patterns;
PC	pancreatic cancer;
PCa	prostate cancer;
PDA	pancreatic ductal adenocarcinoma;
PKR	RNA-dependent protein kinase;
PPARα	proliferator-activated receptor α;
PYD	pyrin domain;
ROS	reactive oxygen species;
SCLC	small-cell lung cancer;
SNPs	single nucleotide polymorphisms;
SKCM	skin cutaneous melanoma;
TAMs	tumor associated macrophages;
TFEB	transcription factor EB;
TLRs	toll-like receptors;
TSLP	thymic stromal lymphoprotein;
ULK1	unc-51 like autophagy activating kinase 1.
